# Correction: An investig-ation into the epidemiology of chikungunya virus across neglected regions of Indonesia

**DOI:** 10.1371/journal.pntd.0010502

**Published:** 2022-06-01

**Authors:** The PLOS Neglected Tropical Diseases Staff

In the title of this article, there is a typographical error in the word “investigation”. The correct title is: An investigation into the epidemiology of chikungunya virus across neglected regions of Indonesia.
