# Supplementary figures and images for: An investig-ation into the epidemiology of chikungunya virus across neglected regions of Indonesia
Source: PLoS Negl Trop Dis. 2020 Dec 21;14(12):e0008934. doi: 10.1371/journal.pntd.0008934 (PMC7785224; doi:10.1371/journal.pntd.0008934)

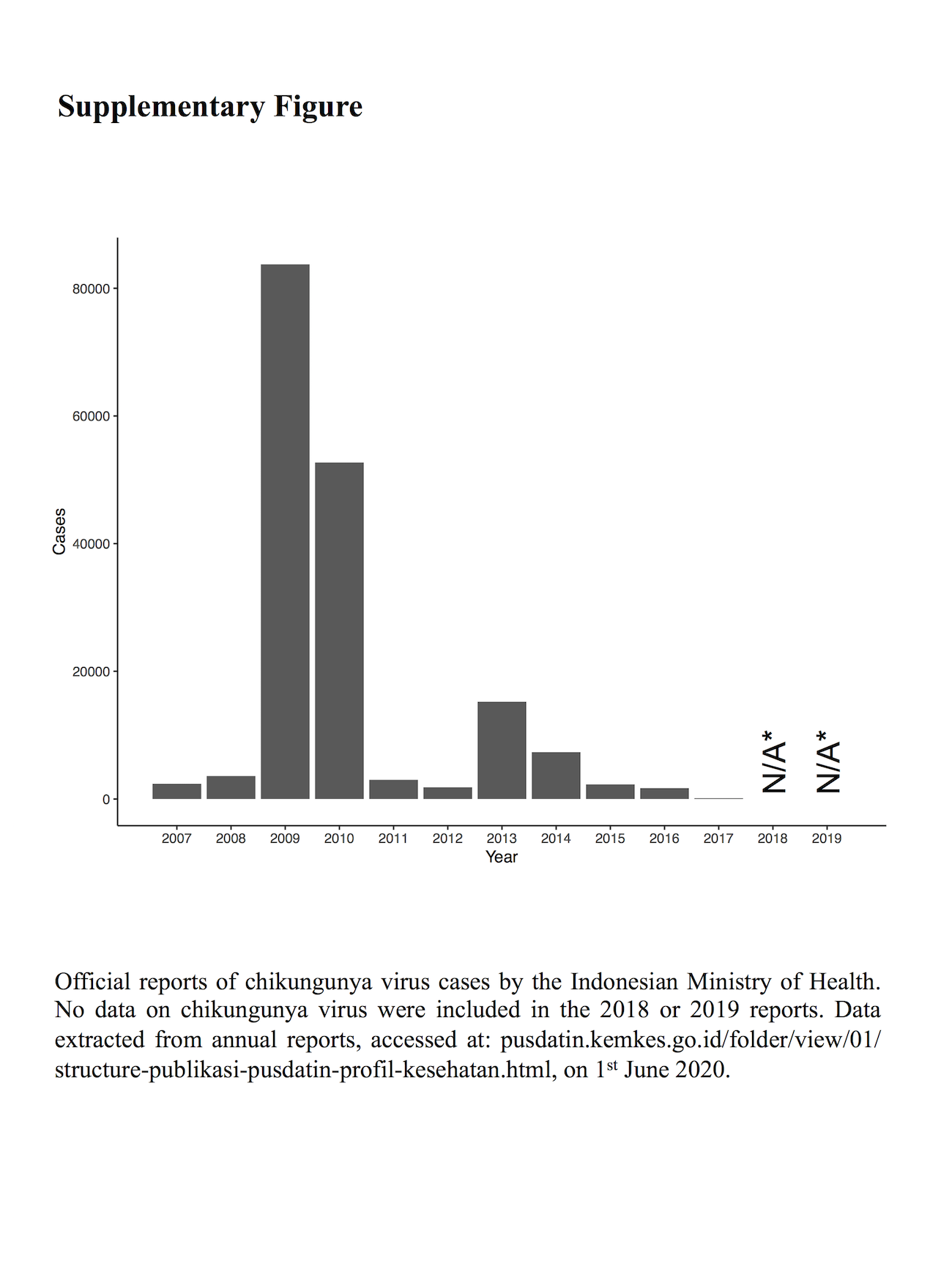

Supplement: S1 Fig — No data on chikungunya virus were included in the 2018 or 2019 reports. Data extracted from annual reports, accessed at: pusdatin.kemkes.go.id/folder/view/01/ structure-publikasi-pusdatin-profil-kesehatan.html, on 1st June 2020. (TIFF) [file pntd.0008934.s002.tiff]
