# Supplementary material for: An investig-ation into the epidemiology of chikungunya virus across neglected regions of Indonesia
Source: PLoS Negl Trop Dis. 2020 Dec 21;14(12):e0008934. doi: 10.1371/journal.pntd.0008934 (PMC7785224; doi:10.1371/journal.pntd.0008934)
Supplement: Attachment — Submitted filename: Response_to_reviewers_25092020.docx [file pntd.0008934.s004.docx]

Author’s responses to Reviewer’s Questions (Responses are in **bold**)

Methods

Reviewer #1: The study aims to characterize the contribution of CHIKV to febrile illness across three cities from Indonesia. The authors had ethical approval to collect samples from symptomatic patients. Despite obtaining 482 samples, the authors were only able to detect CHIKV RNA in only one sample from this recent study-population, besides the detection of IgG in 11 cases. To assess the evolutionary dynamics of CHIKV in Indonesia, the authors performed next-generation sequencing on the single sample identified plus the other 25 archived samples also from previous outbreaks in Indonesia.

Reviewer #2: Yes

Reviewer #3: Line 132-134: Please describe the IgG ELISA and any validation done for the assay. I could find limited description about this in the references (much about IgM assay) and please state here what viral strain/protein was used in the ELISA.

**Response: A reference has been added for the IgG ELISA methodology (Martin et al (2000) J. Clin. Microbiol). Information regarding the viral strain employed by the assay is now also explicitly stated in the text (Lines 155-163). The IgG ELISA assay was developed by CDC and validated collaboration with our collaborators at the Armed Forces Research Institute (Bangkok). However, the validation data has not been published.**

--------------------

Results

Reviewer #1: Results were in general well described and matched the analysis plans. however, it seems there is an inconsistency in lines 231-232 when the authors say that strains from the Indonesia clade were collected between 2007 and 2013 and claim that no further strains from that clade were isolated since 2013. A closer look at the MCC tree box 5 shows an Indonesian isolate sampled in 2014 (MG967260_IDN). Therefore authors should clarify or rectify their statement in the text.

**Response: Thank you, this statement has now been rectified (Lines 288 and 290)**

Reviewer #2: Yes

Reviewer #3: Line 180: It would be helpful to see a figure of the full age distribution of the participants in each place. This is important for interpreting the seroprevalence results. Please comment on the age of the IgG seropositive cases, if there is any pattern with age, then it may be important to age standardise the reported seroprevalences. The paper does however seem to be somewhat skewed towards the results in this section in the main text, with less attention paid to important more detailed analysis/presentation of analysis of the PCR and seroprevalence results.

**Response: Table 2 has been altered to present the age distribution of patients and IgG positive cases in each site. Additional text has been added to the results section to describe these results including the age of IgG seroposititve cases (lines 236 – 249). No statistically significant trend was observed between IgG seroprevalence and age.**

The data availability statement says the data is available, but I could not find the link to this- please add in. If possible this should be list of samples tested with age and then the results of each of the assays. If not possible, grouped into as small age groups as possible.

**Response: Patient age and gender metadata has now been added as supplementary file S2.**

--------------------

Conclusions

Reviewer #1: Conclusions and study's limitations were described, although more data are necessary to support the final statement in the conclusion section (lines 429-430).

**Response: The final statement in the conclusion section has now been amended to reflect this (Line 521).**

Reviewer #2: Yes.

Reviewer #3: Line 331: This comment on age results in the current study needs to be backed up by more presentation of the age seroprevalence results in the text.

**Response: Table 2 has been altered to present the age distribution of patients and IgG positive cases in each site. Additional text has been added to the results section to describe these results including the age of IgG seroposititve cases (lines 236 – 249).**

Line 332: Please expand on the age comparison between current and past studies. Another relevant comparison is between the age groups that would have been included in both studies (but 40 years older in the latest study) and how this compares to your results.

**Response: The discussion has been expanded with regards to age comparison, including a comparison of the age groups that would have been included in the past and present studies (lines 393 – 414).**

Line 333: I’m not at all clear what the < 30 years olds seroprevalence is from past and this study currently. Please clarify.

**Response: Lines 399-402: The seroprevalence for < 30 year olds in both studies has now been clarified in the text.**

Line 340: What is the data you are comparing to from Java and Bali? Even if quoted elsewhere please restate here.

**Response: This sentence is no longer present as the section has been updated as part of the wider response to reviewers.**

Limitations- how do you think the choice of symptoms in subjects could have influenced the PCR and serology results.

**Response: The primary aim of this study was to capture CHIKV viremia, which has been reported to peak at day 0 of symptom onset and persist until day 6, making it unlikely that RNA positive cases were missed by the timing of our sampling approach. However, additional testing of convalescent sera may have resulted in higher seroprevalence rates by allowing time for the IgM and IgG responses to develop.**

**The Asian genotype of CHIKV has been reported to cause relatively mild disease in comparison to other genotypes, particularly in young adults. Therefore, it is possible that CHIKV cases were excluded due to the recruitment criteria and the true prevalence of CHIKV may be higher than reported. However, inclusion of asymptomatic and mild cases was outside the scope of this study, as it would require a dramatic increase in sample size in order to detect active cases of CHIKV infection**.

**Additional text has been added to address these limitations (Lines 483-497).**

--------------------

Editorial and Data Presentation Modifications?

Reviewer #1: (No Response)

Reviewer #2: The manuscript is well written - it could possibly be presented somewhat better by not having so many very short paragraphs, but that is a minor concern.

Reviewer #3: (No Response)

--------------------

Summary and General Comments

Reviewer #1: (No Response)

Reviewer #2: The manuscript is very straightforward, reporting on new Asian genotype sequences of CHIKV as well as a serological study on febrile patients in regional areas of Indonesia.  Studies that add to our understanding of patterns of CHIKV circulation are very valuable, given how neglected this virus is and the sporadic nature of outbreaks.

There is a limitation here in that serological surveys should be much larger in sample size rather than simply opportunistic like this but it is a question of resources.  They should also span a broader range of the population in age, not just febrile patients who overall were very young.  Given that CHIKV has such large intervals between outbreaks, it's possible that a skew towards very young patients is likely to miss serological patterns in older age groups.  I felt that the authors dealt with this limitation relatively well in their discussion, but this could be emphasised.

**Response: As the reviewer states, study size is constrained by resources. The age of recruited patients was skewed towards younger patients but was also relatively broad (6 months – 75 years) and we observed no statistically significant association between seroprevalence and age. However, we acknowledge that, as a consequence of study design, recruitment was not optimal for a seroprevalence survey. We have now emphasised this as a limitation of the study in the discussion (lines 483 – 486).**

Minor comments:

Abstract – lines 30-31: please specify that the archived samples were from outbreaks across the whole of Indonesia so it’s clearer to the reader

**Response: It has now been specified that these outbreaks occurred throughout Indonesia (line 31).**

Line 66: it is misleading to claim the Asian genotype ‘emerged’ in the Americas; a better phrase would be ‘was introduced’.

**Response: Amended as suggested (line 69).**

Lines 132 to 133: were negative PCRs run in duplicate?

**Response: Single PCRs were run due to a limitation in resources, this has now been stated in the methods section (Line 154-155).**

Were there any controls for RNA extraction?

**Response: Yes, an internal extraction control was used and included as part of parallel testing for dengue virus. This has been added to the text (Lines 152 – 154)**

 How do we know these are true negatives?

**Response: RNA extraction controls and positive/negative PCR controls were used to minimise the risk of false negatives due to failed extraction and incorrect PCR reaction set up respectively. Both points have now been added to the text (lines 151-154). We acknowledge that the risk of false negatives could be further reduced by running PCRs in duplicate, however this was not possible due to limitations of resources (both reagents and samples).**

Line 135: binomial confidence intervals for what?

**Response: 95% binomial confidence intervals were calculated for the IgG seroprevalence results. This has been clarified in the text (line 167).**

Lines 166: how were these sequences for Figure 2 chosen?  It does not seem like all Asian genotype CHIKV sequences reported to have an Asian origin were included.  At least it’s not clear if all were included or not.

**Response: The process of sequence selection for Figure 2 is now outlined in the methodology (lines 201-214).**

Line 187: please clarify what was being tested between groups for significance

**Response: Tests were performed to ascertain whether there were statistically significant differences in age and gender ratio between sites. This has now been clarified in the text (Lines 240-241).**

Line 416 – 417: can the authors please provide a reference?

**Response: References added (Line 508)**

The discussion could be a little bit broader in its perspective on the Asian genotype's history in Asia, rather than only focusing on Indonesia.

**Response: Added more detail regarding the Asian genotype’s history in Southeast Asia (Lines 73-77).**

The authors state the sequencing data will be banked at European Nucleotide Archive but I would have expected some provisional accession numbers to be reported. I hope these are provided with any revised version~~.~~

**Response: ENA study number has been added to the text (Line 221).**

Reviewer #3: This an interesting paper, that adds to the picture of chikungunya transmission in Indonesia. The presentation of the seroprevalence results is too brief, and further analysis with regards to age.

**Response: Table 2 has been altered to present the age distribution of patients and IgG positive cases in each site. Additional text has been added to the results section to describe these results including the age of IgG seroposititve cases (lines 236 – 249).**

The discussion of these results in the context of serosurveys from elsewhere is also not sufficiently clear or detailed.

**Response: Expanded on the discussion of seroprevalence results in comparison to previous studies in Indonesia and other Asian countries (lines: 383-423).**

Abstract:

Conclusion: Please state which of your results leads you to conclude low-level endemic transmission.

**Response: ‘Low-level endemic transmission’ has been altered to ‘low-level seroprevalence and molecular detection’ (Line 38).**

Author summary:

I think the statement that this lineage is likely to be introduced is a little strong. Perhaps will lead to imported cases, but whether that will lead to virus introduction, depends on the factors of the countries it is imported to.

**Reponse: The statement has now been amended to state that the lineage has the potential to be introduced (Line 54)**

Main text:

Line 71-73: Please more details on the countries across the region where chik is endemic, and the definition of endemic.

**Response: Added details of Southeast Asian counties where CHIKV is endemic and an explanation of what this means to the text (Lines 79-81).**

Line 90: Suggest more details on where these studies were, what their transmission potential for chik is, and how it was determined that Indonesia was the source of these imported cases.

**Response: Added details of the study locations (Japan and Taiwan), how it was determined that Indonesia was the source (traveller history) and a brief discussion of the transmission potential in these two instances (Lines 100-105).**

Line 91: Same comment as the author summary, consider whether there is sufficient evidence that Indonesia is a source for transmission in other countries.

**Response: This statement has now been amended to only suggest Indonesia is a ‘potential’ source of transmission for other countries (Line 104).**

Line 102-103: Please expand on how it was determined to chose these sites and that they are representing the regions, rather than are in each region.

**Response: These sites are capitals of their respective provinces and are located in three distinct time-zones representing Western, Central and Eastern Indonesia. An explanation of this has been added to the methods section (Lines 131-136).**
